# How Does Weight Loss After Bariatric Surgery Impact the Ocular Parameters? A Review

**DOI:** 10.1007/s11695-023-06607-1

**Published:** 2023-04-27

**Authors:** Krzyżanowska Marta, Czarny Katarzyna, Kroczek Marta, Gniłka Włodzimierz, Michalik Maciej, Kałużny Bartłomiej

**Affiliations:** 1 Division of Ophthalmology and Optometry, Department of Ophthalmology, Collegium Medicum, Nicolaus Copernicus University in Toruń, ul. Ujejskiego 75, 85-168 Bydgoszcz, Poland; 2 Department of General and Minimally Invasive Surgery, Jan Biziel University Hospital Number 2, ul. Ujejskiego 75, 85-168 Bydgoszcz, Poland

**Keywords:** Bariatric surgery, Weight loss, Microcirculation, Intraocular pressure, Vitamin A

## Abstract

Our review aimed to assess the effects of bariatric surgery-induced weight loss on ocular functions. We focused on retinochoroidal microcirculation, glaucomatous factors, and the condition of the eye surface pre- and postoperatively. The review covered 23 articles, including five case reports. Bariatric surgery positively impacts retinochoroidal microcirculation. The arterial perfusion and vascular density improve, venules constrict, and the arteriole-to-venule ratio increases. Weight loss positively correlates with intraocular pressure decrease. The impact of postoperative weight loss on the choroidal thickness (CT) and the retinal nerve fiber layer (RNFL) is still unclear. The correlation between ocular symptoms and hypovitaminosis A needs to be evaluated. Further research is required, especially regarding CT and RNFL, mainly focusing on long-term follow-up.

## Introduction

Excess fat accumulation leads to common health consequences such as cardiovascular diseases, diabetes, and ophthalmic complications. Cataract, age-related maculopathy, diabetic retinopathy, and glaucomatous optic neuropathy are some obesity-related conditions. Early diagnostics and weight loss may stop or reverse obesity-induced changes [1].

This review aims to evaluate the impact of bariatric surgery-induced weight loss on ophthalmic parameters. We decided to focus on three significant aspects that might be useful in everyday practice: intraocular pressure (IOP) and retinal nerve fiber layer (RNFL), retinochoroidal microcirculation, and ocular surface. After bariatric surgery, we investigated the positive and negative ocular consequences of decreased body mass index (BMI).

IOP positively correlates with BMI [2]. Elevated IOP is the leading risk factor for glaucoma and glaucomatous optic neuropathy [3]. Other consequences of obesity are microvasculature alterations and endothelial dysfunction [4]. Retinal microvascular changes reflect the damage from obesity and related conditions and are crucial in obesity-related cardiovascular diseases [5].

Postoperative weight loss and fat malabsorption may cause deficiencies in fat-soluble vitamins [6, 7]. Vitamin A deficiency is especially problematic due to its crucial role in ocular surface and visual acuity [7-9].

## Materials and Methods

We searched the PubMed electronic database from its inception to September 2022 and analyzed the available data on pre- and postoperative ocular changes in patients with extreme obesity. To be included in the study, ocular parameters had to be compared before and after bariatric surgery. We searched for the terms “bariatric,” “bariatric surgery,” “metabolic surgery,” and “gastric bypass” and for combinations of these terms with “ophthalmology,” “ocular,” and “ocular parameters.” After an initial analysis, we narrowed the search to the three most commonly studied areas—retinochoroidal microcirculation, glaucoma risk factors, and ocular surface. We reviewed the reference lists of relevant publications for additional references. The search was limited to English language articles. Studies on ocular surface condition were an exception. Because these changes were observed postoperatively, there were no retrospective studies comparing vitamin A levels before and after surgery. Twenty-three studies were included in this analysis, including five case reports. We presented all relevant data in five comprehensive tables.

## Results

### Retinochoroidal Microcirculation

As the insight into the fundus of the eye enables the examination of the retinal vascularity in vivo, it allows non-invasive observation and follow-up of post-bariatric surgery patients [4]. The results of postoperative changes in retinochoroidal microcirculation are summarized in Table 1.

**Table 1:** Retinochoroidal microcirculation alterations induced by postoperative weight loss

Therapy	Compared to	Measurement	Methods	Number of patients	Follow up (months)	Mean age (years)	Result	Author	Year
GBP, LSG, laparoscopic gastric binding, biliopancreatic diversion	NA	ED, AVR	Digital fundus camera (RVA; Imedos, Jena, Germany), flicker light response	30 (19W, 11M)	6–23	45.3	Improvement in retinal venular widening, AVR, and flicker reaction but no improvement of flicker reaction of retinal arteries	Lammert et al. [15]	2012
GBP in diabetic patients	NA	Retinal thickness, retinal vessels	Digital fundus camera (Visucam Pro NM, Carl Zeiss Meditec, Jena, Germany) SD-OCT (Spectralis, Heidelberg Engineering GmbH, Germany)	51	Up to 12	47.0 ± 8	Increased retinal thickness	Brynskov et al. [22]	2016
LSG	NA	CMT, TMV, SFCT	SD-OCT (Cirrus HD OCT, Carl Zeiss Meditec, Dublin, CA, USA), EDI-OCT	41	6	38.0 ± 8.6	Increased CMT, TMV, SFCT	Dogan et al. [18]	2016
GBP, LSG	Age-matched CG	CRAE, CRVE	Digital fundus camera (Topcon TRC50DX type IA, Tokyo, Japan), SIVA	37 (AT—22, CG—15)	6	AT—43 ± 10; CG—45 ± 12	Increased CRAE, decreased CRVE	Viljanen et al. [30]	2018
GBP	No surgery	FT (average, minimum and maximum value)	SD-OCT (version 1.9.10.0 Heidelberg Engineering, Carlsbad, California, USA)	57 (AT—29, CG—28)	12	46.2 ± 10	Increased minimum FT values in AT	Posarelli et al. [19]	2018
Group A—bariatric surgery, group B—conservative management (exercise/diet)	Age-matched CG	CDI, AVR, retinal thickness, CT, CVI	Digital fundus camera, SS-OCT, SS-OCTA (DRI Triton, Topcon1)	90 (group A—30, group B—30, CG—30)	3	46.47 ± 10.9	Increased CT in group A but not in group B	Agarwal et al. [12]	2020
GBP	NA	Retinal thickness in foveal, perifoveal, and parafoveal regions	SD-OCT (Version 1.10.2.0, Spectralis; Heidelberg Engineering, Heidelberg, Germany)	40 (8 M, 23 W)	6–12	49.4 ± 8.6	Increased foveal, parafoveal, and perifoveal retinal thickness	Laiginhas et al. [20]	2020
GBP	NA	FAZ circularity and perimeter, foveal and perifoveal vascular density in SVP and DVP	Heidelberg Spectralis OCTA system (version 1.10.2.0, Spectralis; Heidelberg Engineering, Germany)	20 (6 M, 14 W)	6–12	50.6 ± 9.0	Increased FAZ circularity and vascular density in perifoveal DVP, decreased FAZ perimeter	Laiginhas et al. [4]	2021
GBP	NA	Macular thickness, macular VD in DVP and SVP	OCT, OCTA (AngioVue system, Optovue RTVue XR Avanti; Optovue, Inc., Fremont, CA, USA)	40 (19 M, 21 W)	3	35.7 ± 3.4	Increased macular thickness in the fovea and parafovea but no significant increase in the perifovea. Increased macular VD in the DVPs but no significant increase in the macular VD in the SVP	El-Shazly et al. [21]	2021
Bariatric surgery	Age-matched CG with normal BMI	Subfoveal, nasal, and temporal CT	SD-OCT (Spectralis, Heidelberg Engineering, Germany), SITA 30-2 (Humphrey Field Analyzer, Carl Zeiss Meditec, Inc., Dublin, CA, USA)	80 (AT—40, 8 M, 32 W; CG—40)	12	38.50 ± 11.14	Decreased CT in subfoveal, nasal, and temporal regions	Gonul et al. [7]	2021
Bariatric surgery	NA	CRVE, AVR	NA	88 (75 W, 13 M)	12	43	Decreased CRVE, increased AVR	Debourdeau et al. [16]	2022

*GBP*, gastric bypass; *LSG*, laparoscopic sleeve gastrectomy; *NA*, not applicable; *ED*, endothelial dysfunction; *AVR*, arteriole-to-venule ratio; *W*, women; *M*, men; *SD-OCT*, spectral-domain optical coherence tomography; *CMT*, central macular thickness; *TMV*, total macular volume; *SFCT*, subfoveal choroidal thickness; *EDI-OCT*, enhanced deep imaging-optical coherence tomography; *CG*, control group; *CRAE*, central retinal artery equivalent; *CRVE*, central retinal venular equivalent; *AT*, active treatment; *SIVA*, Singapore I Vessel Assessment; *FT*, foveal thickness; *CDI*, retinal capillary density index; *CT*, choroidal thickness; *CVI*, choroidal vascularity index; *SS-OCT*, swept-source optical coherence tomography; *OCTA*, optical coherence tomography angiography; *FAZ*, foveal avascular zone; *SVP*, superficial vascular plexus; *DVP*, deep vascular plexus; *VD*, vascular density; *BMI*, body mass index

#### Arteriole-to-Venule Ratio (AVR)

AVR indicates endothelial function and reflects even preclinical metabolic and cardiovascular risk in patients with obesity [10, 11]. AVR changes result from arterial narrowing, venular dilatation, or both. It decreases in the course of weight gain due to increased arteriolar resistance and systemic hypertension [12]. There are various methods to determine the AVR, including mean arteriole and venule width, the sum of widths of arterioles and venules, the sum of squares of widths of arterioles and venules, the central retinal artery equivalent (CRAE), and the central retinal venous equivalent (CRVE) [13, 14].

Digital fundus imaging was used in a study by Lammert et al. A significant dilatation of retinal arteries and decreased retinal vein calibers increased AVR. Moreover, the authors observed a significant increase in adiponectin, regardless of gender [15].

Agarwal et al. demonstrated no difference in AVR between patients with obesity who underwent bariatric surgery or conservative treatment (diet/exercise) [12]. Debourdeau et al. have detected factors predisposing to vein narrowing and increasing AVR. High baseline weight, male sex, and no diabetes history appeared to predispose to more significant improvement of the retinal microvasculature [16].

CRAE and CRVE give more repeatable and accurate results than separate measurements of the width of arterioles and veins of the retina. The SIVA software automatically identifies vessel types and calculates retinal microvascular parameters [5].

#### Choroidal Thickness (CT)

The CT reflects the total choroidal vasculature [12]. Patients with extreme obesity have decreased nitric oxide levels responsible for vasodilatation [17]. Moreover, a positive association between higher BMI and vasoconstrictor factors, endothelin-1 and angiotensin-II, has been found. It may result in a decrease in choroidal blood flow and CT lowering.

A study by Dogan et al. showed a statistically significant increase in subfoveal CT after bariatric surgery [18]. Agarwal et al. considered equal sample size allocation as Dogan et al., which allowed them to reach 95% confidence and 80% power [12]. They observed increased CT in patients with obesity compared to patients with normal BMI, although it did not reach statistical significance. In a study by Gonul et al., a significant decrease in the CT values gave the opposite results. It may result from the patient characteristics, gender distributions, follow up-period, or the OCT manufacturer [7].

#### Retinal Thickness

Postoperative changes in retinal thickness became an object of research in 2019. Posarelli et al. observed an inverse association between minimum foveal thickness (FT) values and BMI. Interestingly, the values of minimum FT remained within the normal limits after bariatric surgery [19].

Research by Laiginhas et al. highlighted that inner retinal layers are more vascularized than the outer layers; therefore, thickening of these layers might reflect the improved perfusion [20, 21].

Laiginhas et al. identified clinical predictors for retinal thickening after bariatric surgery. After the surgery, increased HbA1c and serum C-peptide drop were reportedly associated with retinal thickening in the foveal and parafoveal regions [4]. Brynskov et al., who conducted their study only on type 2 diabetic patients, confirmed that retinal thickness correlates inversely with HbA1c levels [22].

#### Vascular Density (VD)

Optical coherence tomography angiography (OCTA) is a new technology that provides non-invasive imaging and tracking of retinal capillaries. It allows the assessment and detection of retinal microvasculature abnormalities without requiring intravascular dyes in both superficial and deep vascular plexuses (SVP, DVP) and non-perfusion areas [23].

Agarwal et al. were the first to examine changes in SVP and DVP with OCTA before and after bariatric surgery. They obtained the capillary density index (CDI), the percentage of capillary density over the stromal area in a particular region. At the short, 3-month follow-up visit, no significant changes in CDI after surgery were observed [12].

Contrary to the previous study, El-Shazly et al. found a significant statistical increase in macular VD in the DVP 3 months after the bariatric surgery [21]. Their observation agreed with a study by Laiginhas et al., who reported a postoperative increase in perifoveal vascular density in the DVP [4]. Increased perfusion in the DVP is explained by its greater sensitivity than SVP [20].

#### Other Vascular Parameters

Researchers assessed other parameters related to microvascular changes in the eye before and after bariatric surgery. The smaller number of studies disabled the possibility of result comparison. Therefore, these data have been collected in this section.

Agrawal et al. proposed a new imaging tool to indicate the ratio of choroidal vessels to stroma—the choroidal vascularity index (CVI) [24]. CVI reflects the distinction between stromal and luminal vascular components [25]. In another study, Agarwal et al. analyzed the choroidovascular system and capillary density index (CDI), the ratio of the percentage of capillary density to the stromal area in both SVP and DVP. In this study, both groups noted no significant change in CVI and CDI [12].

SD-OCT was used to measure total macular volume (TMV) in the study by Dogan et al. Patients who underwent LSG presented a statistically significant increase in TMV postoperatively [18]. The authors did not explain their methods of TMV assessment, but in a study by Burkholder et al., TMV represented the sum of the volumes of the neural retina in the central 6 mm of the macula [26].

The foveal avascular zone (FAZ) is a circular capillary-free zone in the retina [27]. Microvascular diseases reflect in the increase in size and loss of round contour of FAZ. Increased FAZ circularity and decreased perimeter demonstrate the postoperative improvement in microvascular perfusion in the retina [4].

#### Summary

Higher BMI is associated with narrower retinal arteriolar and wider venular calibers. These microcirculation changes contribute to cardiovascular and cerebrovascular diseases [28]. Examining retinal and choroidal vessels in vivo provides a window to the human microvasculature. It is a non-invasive procedure, does not expose the patient to radiation, and is not associated with side effects.

AVR was the first parameter to be studied in 2012 [15]. With the development of new technologies, it has become possible to explore new links between BMI and microcirculation. OCT quickly became an essential tool in retinal diagnosis, enabling choroidal thickness measurements and vascular density indices. OCT angiography gives an insight into the perfusion in retinal microvasculature. Postoperative AVR improvement, increased choroidal and retinal thickness in the macula, and increased macular VD in the DVP prove that obesity-induced microvasculature alterations might be reversible after weight loss. Current research focuses on advanced technologies, including software for automatic vessel density measurement in DVP and SVP. It may provide even better insight into the subclinical changes in patients with obesity before and after bariatric surgery.

### Glaucoma

*Glaucoma* is an ocular disorder leading to optic neuropathy and the first cause of irreversible blindness worldwide. Increased IOP is a leading risk factor in the pathogenesis of glaucoma. IOP can be elevated mechanically with fat accumulated intraorbital and elevated pressure in episcleral veins. Disrupted vascular homeostasis and endothelial dysfunction are reasons for the vascular etiology of glaucomatous changes. Changes in the RNFL and visual fields are objective parameters used to diagnose and evaluate the progression. In the course of glaucoma, RNFL thinning is noticed during several years of observation [29].

#### IOP

Weight loss is positively correlated with IOP decrease when the surgery succeeds. Postoperative changes in IOP are summarized in Table 2.

**Table 2:** Impact of postoperative body weight loss on intraocular pressure (IOP)

Therapy	Compared to	Measurement	Methods	Number of patients	Follow up (months)	Mean age (years)	Mean IOP before surgery (mmHg)	Mean IOP postoperatively (mmHg)	*p* value	Author	Year
Bariatric surgery	Non-obese age-matched CG	IOP, RE	GAT and PDCT	AT—22 W CG—15 W	6	AT—43 ± 9 CT—45 ± 12	AT: 16.6 ± 3.0 (GAT) 18.1 ± 2.2 (PDCT) CG: 14.3 ± 1.5 (GAT) 16.5 ± 1.9 (PDCT)	AT: 15.2 ± 2.7 (GAT) 16.5 ± 2.0 (PDCT) CG*: 14.5 ± 2.2 (GAT) 16.7 ± 2.3 (PDCT) *—after 6 months	*p*<0.05	Viljanen et al. [30]	2018
LSG	NA	IOP, RE + LE	GAT	32 (24 M)	3–6	40.5 ± 12	17.1 ± 4.0 (RE) 16.8 ± 4.1 (LE)	14.2 ± 2.9 (RE) 14.1 ± 2.9 (LE)	*p*<0.001	Burgansky-Eliash et al. [31]	2018
GBP	NA	IOP, RE+LE	GAT	29	12	46.2 ± 10	15.6 ± 3.5 (RE) 14.9 ± 3.4 (LE)	15.9 ± 2.6 (RE) 16.0 ± 2.6 (LE)	*p*>0.05	Posarelli et al. [19]	2019
LSG, MGB	NA	IOP, LE	GAT	22 (17 W)	12	39.27 ± 11.95	16.68 ± 4.06	13.13 ± 2.23	*p*<0.001	Shimonov et al. [32]	2020

*CG*, control group; *IOP*, intraocular pressure; *RE*, right eye; *GAT*, Goldmann applanation tonometer; *PDCT*, Pascal dynamic contour tonometer; *W*, women; *AT*, active treatment; *LSG*, laparoscopic sleeve gastrectomy; *NA* not applicable; *LE*, left eye; *MGB*, laparoscopic mini gastric bypass; *M*, men; *GBP*, gastric bypass surgery

In a study by Viljanen et al., IOP values significantly decreased postoperatively. Measurements obtained with the Pascal dynamic contour tonometer were higher than with the Goldmann applanation tonometer [30]. Burgansky-Eliash et al. and Shimonov et al. also observed a significant IOP decrease, where it remained reduced at the 1-year follow-up visit [31, 32]. In a study by Posarelli et al., authors demonstrated no compatibility between IOP values 3 months and 1 year postoperatively [19].

#### RNFL

Changes in RNFL after the bariatric surgery are presented in Table 3.

**Table 3:** Postoperative changes in retinal nerve fiber layer thickness (RNFL) measured by spectral domain-optical coherence tomography (SD-OCT Spectralis; Heidelberg Engineering GmbH)

Therapy	Compared to	Number of patients	Follow up (months)	Mean age (years)	Results	Author	Year
Bariatric surgery	Age-matched Non-obese CG	AT—22 W CG—15 W	6	AT—43 ± 9 CG—45 ± 12	No change in RNFL	Viljanen et al. [30]	2018
GBP	NA	29	12	46.2 ± 10	Increased RNFL in superior sector, decreased RNFL in nasal sector	Posarelli et al. [19]	2019
LSG, MGB	NA	22 (17 W)	12	39.27 ± 11.95	Reduction in RNFL	Shimonov et al. [32]	2020
Bariatric surgery	Age-matched Non-obese CG	80 (AT—40, 32 W; CG—40)	12	38.50 ± 11.14	No change in RNFL	Gonul et al. [7]	2021

*CG*, control group; *AT*, active treatment; *W*, women; *GBP*, gastric bypass; *NA*, not applicable; *LSG*, laparoscopic sleeve gastrectomy; *MGB*, laparoscopic mini gastric bypass

Posarelli et al. concluded that blood pressure is inversely related to RNFL thickness, supporting the assumption of vascular pathogenesis of RNFL changes in subjects with obesity [19]. After the bodyweight dropped, systolic blood pressure (SBP) significantly decreased. Postoperatively RNFL thickness increased significantly in the superior sector of both eyes and decreased in the nasal sector in the left eye.

Shimonov et al. presented contrary results. In their study, RNFL thickness was significantly reduced postoperatively. The authors hypothesize that postoperative decrease of fat tissue or vascular changes might impact RNFL [31]. Other authors gaunt no statistically significant differences in RNFL thickness [20, 30].

#### Summary

A positive correlation between IOP and BMI is indisputable. The lower the body weight after the bariatric surgery, the lower the risk of glaucoma. Although the authors agree on the outcome, the mechanism of these findings needs to be investigated. On the contrary, RNFL thickness measurements need to be evaluated in long-term analyses to receive more unanimous results.

### Ocular Surface

#### Ocular Surface Condition

*Dry eye* is a multifactorial disease of the tears and ocular surface that results in symptoms of discomfort, visual disturbance, and tear film instability. The clinical evaluation includes Schirmer’s test I and BUT (break-up time), ocular surface staining through fluorescein and rose bengal, and OSDI (Ocular Surface Disease Index) questionnaire to evaluate subjective symptoms such as sensitivity to light, grittiness, or blurred vision [33].

The ocular surface condition following bariatric surgery is summarized in Table 4.

**Table 4:** Ocular surface condition following bariatric surgery

Therapy	Compared to	Methods	Number of patients	Follow up (months)	Mean age (years)	Vitamin A supplementation after the surgery	Conclusion	Author	Year
RYGB Open (94%), laparoscopic (6%)	NA	Questions related to ocular symptoms followed by eye examination and vitamin A serum level	64 (53 W)	>12	43 ± 9	Multivitamin (~3500 IU of vitamin A) but no additional vitamin A supplementation	Low vitamin A levels and frequent ocular complaints	Eckert et al. [37]	2010
Vertical gastrectomy or RYGB	NA	Schirmer’s test I, TBUT, OSDI, ocular surface staining, confocal microscopy (Heidelberg Retina Tomography 3 - Rostock Cornea Module, Heidelberg Engineering, Germany)	28 (22 W)	6	39.4 ± 12.0	5000 IU of vitamin A supplementation in 67.9% of patients	No impact on serum levels of vitamin A, visual function, or ocular surface	Brandão et al. [34]	2017
Bariatric surgery using the Capella technique—1-12 months after surgery (group 1)	Bariatric surgery using the Capella technique > 12 months after surgery (group 2)	Tear ferning test, TBUT, Schirmer’s test I, OSDI, ocular surface staining, impression cytology	89 (81 W)	<60	Group 1: 43.4 ± 8.1 Group 2: 46.5 ± 7.3	Neglecting the use of vitamin supplements in 92% of patients	No dry eye symptoms	Marques et al. [35]	2017
RYGB (18), single anastomosis gastric bypass (2)	Age- and sex-matched CG	CCM (Heidelberg Retina Tomography 3 with Rostock Cornea Module; Heidelberg Engineering, Germany), NDS and NSP questionnaires	AT—20 CG—22	12	AT—48.8 ± 8.3 CG—54.0 ± 9.4	NA	Improvement in keratocyte density and corneal nerve regeneration after the surgery	Iqbal et al. [36]	2021

*RYBG*, Roux-en-Y gastric bypass; *NA*, not applicable; *W*, women; *IU*, international unit; *LASIK*, laser in situ keratomileusis; *TBUT*, tear-film break-up time; *OSDI*, Ocular Surface Disease Index; *CCM*, corneal confocal microscopy; *NDS*, neuropathy disability score; *NSP*, neuropathy symptom profile; *AT*, active treatment; *CG*, control group

Brandão et al. studied a group of patients who underwent bariatric surgery. Postoperatively, most patients reported mild to severe dry eye symptoms. No statistically significant correlation was found between variables such as visual function, ocular surface condition, and vitamin A serum level. However, some limitations of this study, such as no pre-surgery database, small sample, poor sex diversification, and a relatively short observational period, are noticeable [34].

On the contrary, Marques et al. noticed no dry eye symptoms in bariatric surgery patients. The fact that 20% of patients maintained a BMI > 35 mg/m^2^ postoperatively may have contributed to this [35]. Slater et al. had similar conclusions, with 27.3% of patients with postoperative BMI >35 kg/m^2^ [6].

Iqbal et al. focused on assessing confocal microscopy parameters such as corneal nerve fiber density (CNFD), corneal branch density (CNBD), corneal nerve fiber length (CNFL), and keratocyte density (KD) before and 12 months after bariatric surgery. CNFL, CNBD, and keratocyte density from all three stroma layers were significantly lower in patients with obesity compared to controls. Confocal microscopy parameters significantly improved 12 months after bariatric surgery [36].

Eckert et al. determined seven patients with decreased vitamin A levels (<38 ug/dl) out of 64 patients after Roux-en-Y gastric bypass surgery. They assessed the correlation between ocular symptoms, and vitamin A deficiency was assessed. Six of them presented decreased visual acuity (*p* = 0.05) and xerosis (<0.05), five had night vision deterioration (*p* = 0.08), and four patients suffered from eye pain or foreign body sensation (*p* = 0.16). However, further examination indicated meibomian gland dysfunction as a possible cause of dry eye symptoms [37].

#### Vitamin A Deficiency

Vitamin A is one of the fat-soluble vitamins that cannot be synthesized by the human body and must be ingested to preserve tissue storage. The role of vitamin A is essential for the maintenance of correct vision and ocular surface integrity [34].

The ocular surface evaluation, visual function, and vitamin A deficiency following bariatric surgery are summarized in Table 5. We present several case reports describing the long-term side effects of vitamin A deficiency.

**Table 5:** Visual deterioration, ocular surface, and vitamin A deficiency following bariatric surgery—case reports

Therapy—time from surgery to presentation	Symptoms and duration	Demography (age and gender)	Vitamin A level (normal range): at the presentation—after treatment	Treatment	Result	Author	Year
Gastric bypass—3 years	• BCVA OD=OS 20/800 • Peripheral constriction (Humphrey) • Diffuse conjunctival xerosis with Bitot’s spots • Diffuse corneal punctate keratitis with subepithelial scaring	39 W	5 (38–106)—65 ug/dl	• 10,000 IU daily oral • Preservative free artificial tears every 2 h • Bilateral lower punctal plugs inserted • Topical erythromycin ointment at bedtime	• BCVA OD=OS 20/40 • Persistent conjunctival xerosis • Corneal keratitis resolved (scaring present) • Resolution in visual field test	Lee et al. [41]	2005
RYGB 8 years followed by LASIK 2 years	• VA OD=20/25, OS = hand motion • Corneal ulceration OS • Recurrent severe dry eye OS • Diffuse punctuate epitheliopathy OD • 2–3 months • Decreased color vision an night blindness (2 years)	41 W	<2 (30–95)—13 ug/dl	• 100,000 IU per day • Bandage contact lens OS • Tarsorrhaphy OS followed by topical antibiotic therapy	20/100 (pinhole to 20/50) with a central corneal scar that required penetrating keratoplasty	Donaldson et al. [38]	2012
RYGB—3 years	• BCVA OD 6/36 OS 6/36 • Peripheral constriction (Humphrey) • Xerophthalmia, optic atrophy in ophthalmoscopy • 8 weeks	49 W	0.2 (1.0–3.1)—1.1 umol/l	• 200,000 IU 2 days • 50,000 IU daily/2 weeks i.m.	BCVA OD 6/6 OS 6/9 Almost complete resolution of optic atrophy	Fok et al. [40]	2012
Jejunoileal bypass surgery—18 years	• Night blindness • Dyschromatopsia • Dry eyes • 3 months	57 W	0.8 (1.0–3.1)—0.98 umol/l	• 200,000 IU 3 days • 100,000 every 3 months i.m.	Night blindness and dry eye symptoms resolved	Fok et al. [40]	2012
Several bariatric procedures (stomach stapling, gastric bypass, and subsequent revision of the bypass)—10 years	• VA OD = OS 20/25 • Corneal diffuse punctate • Bitot’s spots • Several months	41 W	3 (38–98)—14 ug/dl	• 8000 IU oral daily per month • Artificial eye drops	• VA 20/20 • Night blindness and dry eye resolved • Corneal staining and Bitot’s spots resolved • Conjunctival punctate staining still present	Crum et al. [39]	2017
RYGB—2 years	• VA OD 1.0 OS 0.6 • Night blindness • Color perception slightly decreased • ERG—decreased • Slight consistent in central visual field (Goldmann) • 12 months	37 M	0.09 (1.05–2.45)—NA	25,000 IU oral per day	Nyctalopia and ERG abnormalities improved	Pless et al. [42]	2019
RYGB—19 years and gastric banding 8 years	• VA OD 0.25 OS 0.16 • Severe dyschromatopsia • Bilateral papilledema • Severe dry eye • Central scotoma • 6 months	48 W	<0.007 (1.05—2.54)—NA	25,000 IU oral per day	• Visual acuity 1.0 OD 1.0 OS • Resolution of dry eye color vision, papilledema, improvement in visual sensitivity, and the size of blind spots	Pless et al. [42]	2019

*BCVA*, best corrected visual acuity; *OD*, *oculus dexter*, right eye; *OS*, *oculus sinister*, left eye; *W*, woman; *IU*, international unit; *RYGB*, Roux-en-Y gastric bypass; *LASIK*, laser-assisted in situ keratomileusis; *VA*, visual acuity; *ERG*, electroretinogram; *M*, man; *NA*, not applicable

Donaldson et al. described the case of a 41-year-old woman who had bariatric surgery 8 years before laser-assisted in situ keratomileuses and then developed corneal ulceration that required combined non-invasive and invasive treatment. The levels of vitamin A were less than 2 ug/dl at the time. Finally, this patient underwent a penetrating keratoplasty because of central corneal scarring [38].

Crum et al. reported Bitot’s spots, an ocular manifestation of a systemic disease due to severe hypovitaminosis A, after bariatric surgery in a woman who presented dryness and diminished night vision symptoms. Given the patient’s history of bariatric surgery, anemia, and vitamin D deficiency, further investigation into micronutrient levels indicated a severe vitamin A deficiency. Oral vitamin A supplementation in this patient resulted in the complete resolution of her symptoms within 2 months [39].

Fok et al. reported two cases of female patients who presented visual deterioration developed due to decreased vitamin A serum levels resulting from ceased or irregular supplementation of vitamin A after bariatric surgery. Intramuscular vitamin A injections and oral multivitamin supplementation significantly improved visual function [40].

Lee et al. presented a case report of a woman who suffered from severe bilateral dry eye and visual deterioration due to inadequate vitamin A supplementation for 18 months following a duodenal switch gastric bypass surgery. Almost complete resolution of symptoms and normalized vitamin A level were achieved 6 months after presentation [41].

#### Summary

Studies on ocular surface disturbances following bariatric surgery have many limitations, including small, poor sex-diversified samples without a control group. In most studies, no significant change in ocular surface parameters was observed. Clinical presentation of vitamin A deficiency may imply many different ocular morbidities. Ocular complications of hypovitaminosis A are potentially reversible; however, in some cases, the recovery was significant but incomplete. Vitamin A supplementation after bariatric surgery is crucial to prevent severe visual impairment. Long-term side effects of insufficient vitamin A supplementation and its influence on the ocular surface and vision are documented mainly by case reports.

## Conclusions

To date, bariatric surgery is one of the most efficient methods to reach a stable body weight. As the BMI decreases, ocular changes occur. Almost all studies agree on the positive impact of weight loss following bariatric surgery on retinal microvasculature. Improved arterial perfusion, retinal venular narrowing, and an increase of AVR occur postoperatively. The thickening of the retina following surgery-induced weight loss is observed mainly in the foveal and parafoveal regions. Positive changes were noticed in vascular density, especially in the macular DVP. All authors agree that weight loss in patients with extreme obesity is conducive to IOP decrease, which lowers the risk of glaucoma.

Nevertheless, knowledge about ocular changes after bariatric surgeries is still limited. The impact of the postoperative weight loss on the thickness of the choroid and RNFL is yet unclear. Results in analyzed studies vary, sometimes due to different techniques of examination, patient characteristics, gender distributions, or follow up-period. Both CT and RNFL outcomes need to be evaluated in further research. There is little evidence that the ocular surface condition changes following bariatric surgery. The authors observed vitamin A deficiency in most of the studies. The correlation between ocular symptoms and vitamin A deficiency needs further assessment.

Discussed studies are not free of limitations. Firstly, they have relatively small sample sizes. Groups of up to 88 patients were examined pre- and postoperatively [16]. Secondly, the study populations were mainly women, so further studies are required to investigate gender differences. Moreover, most of the studies lack a comparator group. A longer follow-up would help assess the durability of ocular changes after bariatric surgery.
